# Follow up to ‘Clinical, microbiological characteristics and predictors of mortality in patients with carbapenemase producing Enterobacterales bloodstream infections: a multicentre study’

**DOI:** 10.1016/j.infpip.2023.100324

**Published:** 2023-11-10

**Authors:** Manjula Meda, Michael Collins, Michael Weinbren

**Affiliations:** a Frimley Health NHS Foundation Trust, Portsmouth Rd, Frimley, Camberley GU16 7UJ, UK; b Chesterfield Royal Hospital NHS Foundation Trust, Chesterfield Rd, Calow, Chesterfield S44 5BL, UK; c New Hospital Programme, NHS England, 10 South Colonnade, Canary Wharf, London, E14 4PU, UK

Dear Sir or Madam,

We read with interest the article by Vanessa Anton- Vazquez *et al.* on the ‘Clinical, microbiological characteristics and predictors of mortality in patients with carbapenemase producing Enterobacterales bloodstream infections: a multicentre study. [1] We thank the authors for their useful contribution to this topic and helping to move the AMS agenda forward.

In assessing antimicrobial therapy three definitions were used;

**effective empirical antibiotic –** administered before antimicrobial susceptibility results are available
**early effective antibiotic** – receipt of at least one *in vitro* active antibiotic within 48 hours of blood culture collection
**effective definitive antibiotic –** administered following susceptibility results, defined later as within three days of blood culture positivity.

Ineffective empirical antimicrobial therapy for BSI (as judged by laboratory testing) has been shown in several studies to be associated with increased mortality. The latest, Kadri *et al.*, looked retrospectively at more than 21,000 BSI in the USA. [2] Approximately 20% received ineffective empirical antimicrobial therapy. This was associated with increased mortality, even in patients without sepsis. The article concluded that early identification of bloodstream pathogens and resistance will probably improve population level outcomes.

How quickly does effective empirical therapy need to be established in patients with BSI? Lee *et al.* looked at the timing of appropriate empirical antimicrobial administration and outcome of adults with community onset bacteraemia. Patients were stratified into mildly, moderately and critically ill subgroups. [2] For patients who were classified as being non-critically ill, effective antimicrobial therapy administration within the first forty-eight hours of presentation was necessary to prevent increased mortality. For critically ill patients’ effective antimicrobial therapy prescribed within an hour was necessary to prevent increased mortality. However even within this group rapid microbiological results may still have an impact on survival of individuals.

How should effective therapy be defined? As effective therapy, judged by patient outcomes, is dependent both upon effectiveness of antibiotic and timing of administration; it also requires a parameter cognisant of both. Administration of an antibiotic with *in vitro* activity too late in the course of infection should not be classed as definitive effective antimicrobial treatment. Secondly, what time standard should be set and what starting point? From a patient perspective it should begin as soon as an infection requiring collection of blood cultures is recognised, so time of blood culture collection is a good starting point (commensurate with the Lee *et al.* publication). Lastly, a further variable that should be taken into consideration when reporting effective system treatment is the patient's clinical condition.

We question the use of the term ‘effective definitive antibiotic’ therapy as defined in the article and feel the definition permits treatment received too late in the course of infection to prevent mortality. It also undermines the fundamental purpose of processing blood cultures – to extract the key information (which may be Gram stain, organism ID or antimicrobial susceptibilities) in the shortest time and to utilise this without delay to guide patient treatment.

To place an exact time definition on institution of effective therapy would need to consider the severity of the patient condition. From Lee's data this should be less than forty-eight hours from presentation (collection of blood culture being a reasonable surrogate marker) for non-critically ill patients. From a laboratory perspective the patient's condition at time of collection is often unknown, but even if known this could deteriorate shortly after blood cultures are collected. Therefore, all blood cultures should be processed while preventing or minimising avoidable delays.

Time from collection of blood culture to loading for incubation, and volume of blood cultured are not only pathology key performance indicators, but also quality indicators of how any organisation manages patients with sepsis. We suggest further KPIs to include the percentage of patients on ineffective empirical antimicrobial therapy, the reason for ineffective therapy and analysis of time taken from blood culture collection to institute antimicrobial therapy with effective *in vitro* activity. Avoidable delays should be measured and reported. The burden of avoidable delays is significant and may exceed 48 hours.

Most positive blood cultures in an optimised pathway will signal positive within 12 hours of collection. [3] The processes and technology exist to obtain the identification and antimicrobial sensitivities of the most common organisms within relatively short periods of time, such that most patients can transfer to effective therapy less than twenty-four hours after blood culture collection. We agree with the authors that the high case fatality rate underscores the urgent need to improve timely diagnosis and treatment approaches.

Lastly, we would like to highlight the importance of the hospital environment, notably water and wastewater, which are not currently recognised in the AMR national action plan, but are significant and more importantly preventable sources of transmission of multi-drug resistant organisms. [4] The landscape has changed, and UK hospitals are no longer only seeing imported CPE infections. Guidance to stem the risks caused by the built environment is urgently required in UK hospitals to prevent transmission of multi resistant Gram-negative organisms including CPEs.

## Conflict of interest

None declared
